# Home bias and local equity portfolio decisions of Chinese insurance institutional investors

**DOI:** 10.1371/journal.pone.0288250

**Published:** 2023-07-14

**Authors:** Shanshan Yang, Na He, Haizong Yu

**Affiliations:** 1 Business School, Chengdu University, Chengdu, China; 2 Business School, Southwest Minzu University, Chengdu, China; 3 Accounting School, Southwestern University of Finance and Economics, Chengdu, China; Massey University - Albany Campus: Massey University - Auckland Campus, NEW ZEALAND

## Abstract

Our paper explores whether Chinese insurance companies exhibit a local preference in deciding their equity portfolios and the incentive of this preference. Our research finds that Chinese insurance institutional investors significantly tilt to invest in local firms geographically close to them, and local investments do not significantly outperform non-local investments. The results indicate that the behavioral aspect of home bias, rather than information advantage, play a more significant role in deciding equity holdings of China’s insurance companies. Additionally, local equity preference is more pronounced in dialect-segmented areas and life insurance firms. This paper incorporates hometown identification into the analysis framework of insurance companies’ portfolio decision-making and enriches the research of their investing strategies from the perspective of behavioral finance.

## 1. Introduction

With the fast growth of China’s insurance industry since 2003, the available balance of insurance capital has rapidly developed from 0.57 trillion yuan in 2004 to 16 trillion yuan in 2018, increasing at an average annual growth rate of 22.4% from 2004 to 2017 [1]. Except for its fundamental role in providing insurance products, the insurance industry plays an immensely vital role through its investments in equity and fixed income securities. In the first half of 2018, insurance companies in China appeared as the Top-10 tradable shareholders of 391 Chinese listed companies, becoming the second-largest institutional investors in China’s public capital market. Historically, insurers have been relatively conservative, buy-and-hold investors, with the investment of premiums funding later payments of policyholders; thus, it is conceivable that their equity portfolio decisions are not as radical as that of other institutional investors, e.g., mutual funds. It proves why the equity investments of insurance companies show resilience in stabilizing financial markets at a macro level in the long run [2]. However, the annualized rate of return on portfolio investments of insurance capital in China is about 4.5% in 2018, with a decreasing rate of return on investments year by year. Several studies have argued that the contradiction between the rapid growth of premium income and the low efficiency of insurance funds’ return on investment has become increasingly prominent, attributing to restrictive investment channels and the inability and inadequacy to diversify investment allocations [3]. Local equity preference is perennial worldwide among institutional investors [4]. Our paper aims to research the efficiency and decisions of equity investments of insurance companies and the incentives that drive equity portfolio managers involving local investing in such investment behaviors. Moreover, since January 2019, Chinese Banking and Insurance Regulatory Commission has released positive signals for giving insurance capital more quotas in holding shares of Chinese A-share high-quality listed firms with an approach to be strategic investors; thus, this gives the Chinese insurance sector a powerful and intriguing setting to study the causes and consequences of investment behaviors in insurance equity holdings.

Investors should diversify their holdings across international equity markets [5, 6]. However, this contradicts with institutional managers investing in what they know: the local geographical preference in securities holdings is perennial worldwide. Prior studies have found that, in cross-border equity markets, corporate equity is essentially held by domestic investors [4, 7]; and in domestic equity markets, the stocks of domestic firms are more often held by investors nearby [8, 9]. Additionally, both individuals and professional fund managers exhibit geographical preferences in picking their portfolio allocations [10]. The literature on the geography of investments revolves around different motives of why investment portfolios are biased towards domestic securities. Coeurdacier and Rey (2013) review various explanations of the local preference puzzle: (i) motives for hedging in frictionless financial markets, (ii) transaction costs decreased due to geographical superiority, and (iii) investing optimism and behavioral biases [11]. Besides, some papers link explicit barriers to international investment [12, 13]; and some studies on corporate governance and home equity preference [14, 15]. By far, the motives that lead to local preference of investors are driven by information advantages or behavioral bias is a subject of debate in various strands of literature [8, 10, 16, 17]. Geography and distance effect has an important impact for the Chinese stock market [18]. Despite the rapid growth of the insurance sector, there is relatively little understanding of how insurance equity managers decide their equity portfolios and, in turn, how their choices affect the underlying funds’ efficiency; thus, our paper fills this gap by explicitly analyzing the geographical preferences when China’s insurance companies choose equity investment allocations. Having a better knowledge of the investment strategies of insurance funds will help us better understand the investment behavior of insurance equity managers and the performance efficiency and motivation of the underlying insurance funds.

We start by investigating whether Chinese insurance companies exhibit a local preference in deciding their equity portfolios. We take China A-share non-financial listed firms during the 2006–2016 period as the research sample and construct a data structure of “insurer-firm-year” to examine the relationship of geographical proximity between insurers and their security holdings. Chinese data provides a positive policy setting as the Chinese government has continuously promoted and expanded the size of equity investments for insurance capital. To ensure the results are robust, we use three types of measures to proxy the geographical proximity. We find that insurance companies, on average, overweigh firms nearby to a substantial degree. We next test whether the local preferences of insurance investors generate better performances. We then find that local investments do not significantly outperform other non-local investments. The results found in these two steps show that local preferences badly affect the investment return of funds’ equity portfolios, which supports the familiarity hypothesis. We also document a significant cross-sectional variation in the local equity preference, indicating that the degree of local preference is related to several characteristics of insurance companies in a plausible way. For example, insurance funds from culturally segmented areas invest more heavily in local firms. Additionally, life insurers tilt more local holdings than non-life ones.

This study contributes to the literature on the determinants of local preferences of institutional investors by examining the phenomenon of "local preference" in Chinese insurance investors equity holdings. Li (2004) found investors tend to invest in the stocks headquartered in the fund manager’s home country [19]. Huberman (2001) presents that people often invest in the familiar [16]. Hasan and Simaan (2000) prove that the nature of the estimation risk in international investing causes domestic investing to dominate over international diversification [7]. As highly regulated institutional investors, insurance firms act more professionally in planning investment portfolio decisions than individual investors, and they also are less affected by personal attitudes and psychological factors [10, 20]. Thus, focusing on the geographical preference of insurers’ equity investment provides a more powerful setting for demonstrating homes bias acts as one of the determinants driving local preferences.

Secondly, this paper broadens the research on the impact of behavioral factors on investment performance. We examine the investment performance of institutional insurance investors due to home bias. We assume that insurance equity managers keep informative in utilizing local networks when making equity portfolios decisions; however, the underperformance reminds us that because investors are not sophisticated enough and financially savvy in their local preferences, there is a risk of damaging stakeholders’ benefits.

Lastly, the paper contributes to the role of culture in affecting institutional strategies. We document that the degree of local equity preference varies with several characteristics of insurance companies in a plausible way. It is found that insurance companies particularly insurers located in dialect-segmented areas and life insurers have the home bias, which is complementary and consistent with behavioral explanations of the importance of personal values and experiences on shaping various investment strategies [10, 16, 21].

The remainder of the paper is organized as follows. Section 2 develops testable hypotheses by introducing the theoretical background and hypotheses of whether and why insurance firms are biased to equity portfolio decisions. Section 3 specifies research designs and describes the sample and measures. Section 4 presents the main empirical results of how familiarity affects firms’ portfolio decisions using two steps. Besides, this section also documents abundant cross-sectional tests and robustness tests. Our conclusions and policy implications are given in section 5.

## 2. Theory background and hypothesis development

Among most of the literature studying the motive for local investing, whether the local investing is driven by information superiority or familiarity bias is a subject of debate in the literature [8, 10, 21]. Prior studies have observed that many investors tilt their portfolios nearby [10, 16, 17, 22–24]. Kang and Kim (2008) hypothesize that such preference arises due to the reduced cost of information collection and monitoring that comes with geographical proximity [25]. Furthermore, insurance companies face information asymmetry due to limited disclosure requirements and regulatory constraints, which necessitate close scrutiny of investment targets and post-investment monitoring. More specifically, insurance equity managers work under a relatively more restrictive set of regulatory constraints, and their strategies are monitored and evaluated under the aegis of the “prudent man rule” [20]. Although the increasing power of information technology and transportation globalization has considerably lowered down barriers to communication, it is worth noting that many private information is still not publicly be disclosed in equity investment markets and is often easier to spread through informal channels (e.g., social networks). Despite technological advances and globalization, private information remains difficult to access publicly, and investors may rely on local networks and social channels to gather sensitive information for investment decisions [26, 27]. In this way, geographic proximity enables lower transaction costs associated with communication and access to investee targets, facilitating easier information transmission [22, 28]. These findings shed light on the underlying information advantage for local investing preferences in literature.

Alternatively, the local equity preference of investors may stem from a positive sentiment about their local investing accompanied by a negative attitude towards foreign investing [4, 29] without real information about them [10]. Strong and Xu (2003) find that fund managers from the United States, the United Kingdom, continental Europe, and Japan favored their home equity market [30]. Several studies focus on behavioral explanations, not the institutional factor, for the local preference [31–33]. For instance, Solnik and Zuo (2012) link investors’ regret and disgust in investment decisions to “home bias” when foreign investing underperforms domestic investing [31]. Managers feel connections with these familiar firms, giving them subjectively perceived information advantages, which may lead to the preference for stocks headquartered nearby. However, Huberman (2001) suggests that such perceived information superiority can lead to negative consequences, reminding that the presumption of home bias exists [16].

In light of the above competing arguments, we develop the following hypothesis.

H1: Insurance companies overinvest in stocks headquartered where the insurance company is currently located, with a higher stockholding ratio.

If Hypothesis 1 is verified, it indicates that insurance companies deliver a “local preference” in their equity investment decisions by investing in companies located geographically nearby. However, whether such proximity-based investment preferences are rooted in information superiority or home bias is still uncertain and can have opposite effects on performance outcomes [10]. On the one hand, distance still hinders the transmission of information among financial market participants in the case of some “soft” information [34]. The information superiority rationale suggests that geographic advantages facilitate personal information, access to investee firms, and the chances of face-to-face communications with the management, enabling investors to make better portfolio decisions by allocating appropriate resources. Secondly, investors can strengthen the degree of supervising holding companies and actively participate in corporate governance to improve the operating performance of listed firms, promote the rise of stock prices, and obtain investment returns. Ivkovi´c and Weisbenner (2005) find evidence for the information hypothesis for individual investors’ money management using stock returns as the performance measure. Fund managers earn higher returns by involving social networks and connections based on their college attendance [35]; thus, funds benefit from local advantages [36].

On the other hand, several studies find limited support for the notion that local holdings lead to better performance outcomes. Seasholes and Zhu (2010) find evidence contrary to the information advantage presumption by employing different performance metrics. Tesar and Werner (1995) exclude transaction costs on foreign markets as justification for local preference by reporting a high turnover rate in the international market compared to the turnover in the domestic market [37]. These contradictory findings can be explained by homophily, optimism, and a sense of identity in familiar settings [4, 10, 24, 38]. Such familiarity may result in less-known stocks, restricted access to information, and a diminished role for institutional monitoring and corporate governance. Therefore, local holdings chosen solely based on investors’ home bias are unlikely to outperform non-local holdings, potentially undermining the effects of geographic advantages.

Therefore, we proceed in the next step to investigate the role of the information hypothesis or the psychological hypothesis work on portfolio decisions. We then ask whether these choices reflect familiarity by examining the performance implications of local investing.

H2a: Local investments held by insurance companies outperform other non-local
investments.
H2b: Local investments held by insurance companies underperform other non-local
investments.

## 3. Data, measures, and methodology

### 3.1. Data

All Chinese non-financial firms listed in the A-share market during the 2006–2016 period constitute the sample of this paper. The decline of stocks in the Chinese capital market in 2018 dragged down the market value of institutional investors’ shares and exposed the risk of a stock pledge. To reduce the impact of external environmental factors, we include all Chinese non-financial firms listed in the A-share market during the 2006–2016 period to constitute the sample of this paper. We eliminate special treatment(ST) firms and samples with missing values for financial data. The data of equity holdings of insurance companies were obtained from the document entitled “Top-Ten Shareholders” and the document entitled “Institutional Investors” in the China Stock Market and Accounting Research (CSMAR) database. As for identifying the local investment of insurance funds, we first refer to the registered place from each listed firm’s prospectus and then manually sort out the geographical information of each insurance firm based on the China Insurance Yearbook (2006–2016). Considering that the equity portfolio decision is probably either made from the headquarter of each insurance company or its branch, we locate the insurance companies at the city level. Other financial statements data, corporate governance data, and other variables are from the CSMAR database. Lastly, we adopt the existing research method of processing cross-border M&A data based on Ahern et al. (2015) [39] and Siegel, Licht, and Schwartz (2011) [40] and the method of process institution-firm data based on Kuchler (2022) [41]. Thus, we construct a data structure of “insurer-firm-year”. For example, the data “China Life Insurance-Vanke-2006” presents whether China Life Insurance held shares of Vanke Co. in 2006 and the shareholding ratio. Finally, a sample comprising of 336825 “insurer-firm-year” observations was created. The statistical software used in this study was Stata 15.0.

### 3.2. Methods and measures

We use the following regression specification to investigate the our first prediction:

$$Insurer_{i,j,t}=\alpha_{0}+\alpha_{1}Local_{i,j,t}+\alpha_{j}Controls_{i,j,t}+\varepsilon_{i,j,t}$$ (1)

where i,j,t index insurers, firms, and years, respectively. We focus on two forms of the measure to proxy the equity holdings of insurance companies (*Insurer*_*i*,*j*,*t*_): *Insurer Dummy*_*i*,*j*,*t*_ and *Holding Ratio*_*i*,*j*,*t*_. *Insurer Dummy*_*i*,*j*,*t*_ is an indicator variable that equals one if insurance company_*i*_ is one of the top ten shareholders of the listed firm_*j*_ in year_*t*_. *Holding Ratio*_*i*,*j*,*t*_ represents the shareholding ratio of the insurance companies_*i*_ as the Top-10 shareholders of the firm_*j*_ in year_*t*_. We proxy the geographical proximity using an indicator dummy, *Local*_*i*,*j*,*t*_, as our main test variable, by taking the value of 1 if the insurance company and the registered place of the stockholding firm are located in the same city; otherwise, it is zero. If insurance companies tilt their investment portfolios toward them, we should find that *α1* is positive and significant. In robustness tests, we adopt two other forms of alternative indicators to proxy the geographical proximity. Our first alternative proxy, *Local province*_*i*,*j*,*t*_, is an indicator dummy that equals to one if the insurance company and the firm are located in the same province; otherwise, it is zero. Referring to Kuchler (2022)’s method, our second alternative proxy, *Ln(distance)*_*i*,*j*,*t*_, is expressed as the natural logarithm of the linear distance between the insurance company and the latitude and longitude of its investee firm’s registration place. Regression (1) is estimated using Poisson Pseudo Maximum Likelihood (PPML) to account for the censoring of investments at zero [41].

In Eq (1), a vector of relevant control variables to mitigate correlated omitted variable issues is included. We include stockholding firms’ financial characteristics such as financial leverage (*Leverage*), size (*Size*), growth (*Growth*), profitability (*ROA*), the dividend payout ratio (*Dividend ratio*), and the ratio of tradable shares (*Free float*). We specifically include a dummy variable to indicate whether the firm is government-controlled (*SOE*), a variable representing the ownership concentration (*Top1*), and the Fan, Wang, and Ma’s (2011) China marketization index (*Market index*) of provinces in which the holding firm’s registered place is located [42]. We also control for several firms’ operating characteristics such as firms’ risk-taking capability (*Risk-taking*) and information opacity proxied by earnings management (*EM*), computed as the absolute value of the residuals of regression using modified Jones model at the industry-year level. Lastly, we control several variables to indicate the level of economic development in which the firm’s registration place is located (*GDP*; *per capita GDP*).

In the next step, we examine whether insurance companies’ local preferences are related to their performance; thus, the following equation shows the OLS regression for our second prediction:

$$Perf_{j,t}=\beta_{0}+\beta_{1}Local\;Shareholder_{j,t}+\beta_{j}Controls_{j,t}+\theta_{j,t}$$ (2)

where j and t index firms and years, respectively. For the investment performance of insurance companies (*perf*_*j*,*t*_), we mainly measure the average return on asset (*ROA1*_*j*,*t*_, *ROA3*_*j*,*t*_) of holding firms in the next year and the next three years, respectively, and the average stock return (*BHR1*_*j*,*t*_, *BHR3*_*j*,*t*_) of holding firms in the next year and the next three years, respectively. *Local Shareholder*_*j*, *t*_ is a dummy variable: If there is at least one local insurance company among the Top-10 shareholders of the listed firm *j* in year *t*, it is assigned a value of 1; otherwise, 0 is taken. It should be noted that to compare the impact of local investments of insurance funds on corporate performance, we excluded samples from the top ten shareholders of listed firms that do not have insurance companies. The definitions of all other control variables are consistent with those of Eq (1). All the detailed definitions and descriptions of each variable are shown in Table 1.

**Table 1:** Definitions of variable. This table provides a detailed and definition and description of the procedures used to construct each variable used in our test analyses. All continuous variables are winsorized at 1% and 99% of the distribution to eliminate any effects caused by outliers.

Variables		Definitions
Dependent variables	*Insurer Dummy*_*i*,*j*,*t*_	An indicator variable that equals one if insurance company *i* is one of the top ten shareholders of the listed firm *j* in year *t*; otherwise, it is zero.
	*Holding Ratio*_*i*,*j*,*t*_	the shareholding ratio of the insurance companies *i* as the Top-10 shareholders of the firm *j* in year *t*
	*ROA1*_*j*,*t*_	Return on asset, computed as the ROA of the listed firm shareholding by the insurance company in next year.
	*ROA3*_*j*,*t*_	Return on asset, computed as the average ROA of the listed firm shareholding by the insurance company in next three years.
	*BHR1*_*j*,*t*_	Stock return, computed as the stock return rate of the listed firm shareholding by the insurance company in the next year
	*BHR3*_*j*,*t*_	Stock return, computed as the average stock return rate of the listed firm shareholding by the insurance company in the next three years
Independent variables	*Local*_*i*,*j*,*t*_	Indicator for whether the insurance company and the registration place of its stockholding firm are located in the same city.
	*Local province*_*i*,*j*,*t*_	Indicator for whether the insurance company and the registration place of its stockholding firm are located in the same province.
	*Ln (distance)*_*i*,*j*,*t*_	the natural logarithm of the linear distance between the insurance company and the latitude and longitude of its investee firm’s registration place.
	*Local Shareholder*_*j*,*t*_	A dummy variable that is 1 if there is at least one local insurance company among the Top-10 shareholders of the listed firm* j* in year *t*
Control variables	*SOE*	Indicator for firms that are ultimately controlled by government
	*Size*	Size of the firm, expressed as the natural logarithm of total assets at year-end
	*Leverage*	Leverage, measured as total debts scaled by total assets
	*Top1*	Ownership concentration, expressed as the shareholding ratio of the largest shareholder of the firm
	*ROA*	Profitability, measured as the net profit divided by total assets at year-end.
	*Growth*	Growth, measured as the change in operating revenue between the current period and the previous period, divided by operating revenue in previous period.
	*Dividend ratio*	total dividends paid scaled by net income
	*Free float*	the proportion of the firm’s tradable shares, measured as tradable shares divided by the total number of share capital
	*Risk taking*	Risk-taking capability, measured as the logarithm of the variance of the annualized monthly stock return rate
	*EM*	Information opacity, proxied by earnings management and measured as the absolute value of the residuals of regression at industry-year level using modified Jones model.
	*Market index*	The natural log of marketization index of China’s provinces in which the firm’s registered place is located according to Fan et al.’s (2011) [42].
	*GDP*	Economic development, measured as the natural log of GDP (RMB yuan) of the city where the firm is located.
	*Per capita GDP*	Measured as natural log of per capita GDP (RMB yuan) of the city where the firm is located.

### 3.3. Descriptive statistics

Table 2 provides a summary of descriptive statistical results for key variables used in the analyses. For the paired data structured by “Insurer-firm-year”, there are 1.61% of stockholding firms have one or more insurance companies as their Top-10 shareholders (*Insurer dummy*); and the average shareholding ratio of insurance companies as the Top-10 shareholders of the stockholding firms is 0.73% (*Holding Ratio*). In terms of the geographical proximity for the paired data, 6.47% of the insurance companies and their holding stocks are located in the same city (*Local*); and 8.24% of them are located in the same province (*Local province*); the average linear geographical distance between the insurance companies and their investee firms is 716.87 kilometers(e^6.5749^). In terms of control variables, 45.91% of investee firms are state-owned enterprises, the mean value of *Leverage* and *ROA* of investee firms are 45.55% and 4.74%, respectively. The descriptive statistical results of other variables are shown in Table 2.

**Table 2:** Descriptive statistics of key variables.

Variables	Mean	SD	P25	P50	P75	N
*Insurer Dummy*	0.0161	0.1260	0.0000	0.0000	0.0000	336,825
*Holding Ratio*	0.0073	0.0580	0.0000	0.0000	0.0000	336,825
*ROA1*	0.0477	0.0478	0.0203	0.0402	0.0707	5,404
*ROA3*	0.0441	0.0449	0.0179	0.0375	0.0649	3,749
*BHR1*	0.1837	0.6148	-0.2076	0.0122	0.3853	5,390
*BHR3*	0.1914	0.3405	-0.0402	0.1159	0.3270	3,733
*Local*	0.0647	0.2459	0.0000	0.0000	0.0000	336,825
*Local province*	0.0824	0.2750	0.0000	0.0000	0.0000	336,825
*Ln (distance)*	6.5749	1.3220	6.4605	6.9798	7.2895	336,825
*SOE*	0.4591	0.4983	0.0000	0.0000	1.0000	336,825
*Size*	21.9909	1.2666	21.1094	21.8441	22.7195	336,825
*Leverage*	0.4555	0.2116	0.2932	0.4556	0.6149	336,825
*Top1*	0.3545	0.1523	0.2324	0.3349	0.4620	336,825
*ROA*	0.0474	0.0413	0.0173	0.0368	0.0645	336,825
*Growth*	0.2508	0.6575	-0.0072	0.1249	0.3010	336,825
*Dividend ratio*	0.2616	0.3137	0.0000	0.1951	0.3587	336,825
*Free float*	0.6460	0.3468	0.4170	0.7127	0.9991	336,825
*Risk-taking*	0.0295	0.0339	0.0097	0.0179	0.0340	336,825
*EM*	0.0735	0.0921	0.0206	0.0462	0.0905	336,825
*Market index*	2.0128	0.2549	1.8601	2.0575	2.2328	336,825
*GDP*	28.5011	0.7917	28.0396	28.5390	29.0870	336,825
*Per capita GDP*	10.7910	0.5334	10.4537	10.8736	11.1999	336,825

## 4. Empirical results and analysis

### 4.1. Correlation analysis of key variables

Table 3 reports the results of the correlation analysis of the main variables. Firstly, the geographical proximities (*Local/Local province*) are significantly positively correlated with the equity holdings of insurance companies (*Insurer Dummy/Holding Ratio*), which preliminarily indicates that it is more likely for insurance companies to overweigh local firms that are geographically nearby, with a higher shareholding ratio. Secondly, the farther the linear distance between the insurance company and the stock’s registration place (*Ln(distance)*), the less likely the insurance company to invest in such firms (*Insurer Dummy*), and the lower the stockholding ratio of the company held (*Holding Ratio*). The above evidence supports the “local equity preference” hypothesis in insurance companies’ portfolio decisions. Lastly, the correlation coefficients of the main variables are not high, indicating that there is no severe multicollinearity between the variables.

**Table 3:** Correlation analysis of key variables.

Variables	(1)	(2)	(3)	(4)	(5)	(6)	(7)	(8)	(9)	(10)	(11)
(1) *Insurer Dummy*	1										
(2) *Holding ratio*	0.98***	1									
(3) *ROA1*	-	-	1								
(4) *ROA3*	-	-	0.88***	1							
(5) *BHR1*	-	-	0.17***	0.15***	1						
(6) *BHR3*	-	-	0.12***	0.22***	0.46***	1					
(7) *Local*	0.02***	0.02***	-0.01	-0.00	-0.02	-0.04	1				
(8) *Local province*	0.02***	0.02***	-0.01	0.00	-0.02	-0.0	0.87***	1			
(9) *Ln (distance)*	-0.02***	-0.02***	0.01	-0.01	0.01	0.05	-0.77***	-0.79***	1		
(10) *SOE*	0.03***	0.03***	-0.08***	-0.10***	-0.01	-0.16***	0.04***	0.01***	-0.02***	1	
(11) *Size*	0.07***	0.06***	-0.10***	-0.14***	-0.06	-0.17***	0.05***	0.04***	-0.05***	0.30***	1
(12) *Leverage*	0.01***	0.01***	-0.38***	-0.43***	0.02	-0.10***	-0.02***	-0.03***	0.01***	0.25***	0.38***
(13) *Top1*	0.03***	0.02***	0.09***	0.05***	0.05***	-0.05***	0.04***	0.03***	-0.05***	0.24***	0.26***
(14) *ROA*	0.02***	0.02***	0.77***	0.74***	0.05***	0.03*	0.03***	0.03***	-0.02***	-0.11***	-0.09***
(15) *Growth*	-0.01***	-0.01***	0.06***	0.03*	-0.02	-0.04**	0.00***	0.00	0.00***	-0.06***	0.03***
(16) *Dividend ratio*	0.01***	0.01***	0.06***	0.05***	0.04***	0.06***	0.00	0.01***	-0.01***	-0.05***	0.06***
(17) *Free float*	0.01***	0.01***	-0.02	-0.04***	-0.00	-0.06	-0.01***	-0.01***	-0.00**	0.17***	0.01***
(18) *Risk taking*	-0.03***	-0.03***	0.01	0.02	-0.03*	-0.02	-0.02***	-0.02***	0.03***	-0.06***	-0.25***
(19) *EM*	-0.02***	-0.01***	0.01	0.00	-0.03*	-0.02	-0.00	-0.00**	-0.00	-0.05***	-0.08***
(20) *Market index*	-0.00	-0.00	0.02*	0.08***	0.03**	0.11***	0.16***	0.19***	-0.22***	-0.17***	0.02***
(21) *GDP*	0.00*	0.01***	-0.01	0.02	-0.10***	0.09***	-0.00***	0.06***	-0.08***	-0.26***	0.05***
(22) *Per capita GDP*	0.01***	0.01***	-0.05***	-0.02	-0.16***	0.01	0.23***	0.23***	-0.25***	-0.18***	0.16***
Variables	(12)	(13)	(14)	(15)	(16)	(17)	(18)	(19)	(20)	(21)	(22)
(12) *Leverage*	1										
(13) *Top1*	0.06***	1									
(14) *ROA*	-0.30***	0.06***	1								
(15) *Growth*	0.07***	0.03***	0.13***	1							
(16) *Dividend ratio*	-0.18***	0.09***	-0.03***	-0.11***	1						
(17) *Free float*	0.15***	-0.07***	-0.05***	-0.14***	-0.04***	1					
(18) *Risk taking*	0.06***	-0.06***	0.12***	0.14***	-0.16***	-0.05***	1				
(19) *EM*	0.11***	-0.01***	0.14***	0.25***	-0.11***	-0.04***	0.19***	1			
(20) *Market index*	-0.13***	0.00***	0.05***	-0.03***	0.09***	-0.13***	-0.10***	-0.03***	1		
(21) *GDP*	-0.16***	-0.04***	0.03***	-0.04***	0.10***	-0.11***	-0.11***	-0.04***	0.66***	1	
(22) *Per capita GDP*	-0.16***	0.01***	0.02***	-0.03***	0.10***	-0.11***	-0.12***	-0.02***	0.68***	0.60***	1

*Local/Local province* are significantly positively correlated with *Insurer Dummy/Holding ratio*, which preliminarily indicates that insurance companies overweigh local firms that are more geographically nearby, with a higher shareholding ratio. *Ln(distance)* is significantly negatively correlated with *Insurer Dummy/Holding Ratio*, which indicates that the farther the linear distance between the insurance company and the stock’s registration place, the less likely the insurance company is to invest in such firms, and the lower the stockholding ratio of the company held.

### 4.2. Regression analysis of the “local equity preference” of insurance companies

The results of the coefficient estimates from various forms of Eq (1) are shown in Table 4. We first aim to explore whether insurance companies’ portfolio weights toward local stocks; therefore, region- (city-level), industry-, and year- fixed effects are added in all regressions to control systemic bias effects. In Table 4, columns 1 and 2 report results when *Insurer Dummy* proxies the insurance company equity holdings, and columns 3 and 4 report results when this measure is proxied by *Holding Ratio*. Columns 1 and 3 are results of only including *Local* and a constant in the regression, and columns 2 and 4 including control variables. Across all specifications in Table 4, the coefficient estimates for *Local* are positive and statistically significant at the 1% level. These coefficients indicate that the geographical proximity is positively associated with (i) the likelihood of the insurance company being large shareholders in firms and (ii) the ratio of the insurance companies holding shares in firms. As shown in column 2, it shows the likelihood of insurance companies investing in local firms is 33.03% higher than that of investing in nonlocal firms, bearing meaning for statistic and economic significance. Overall, the results in Table 4 support our hypothesis that insurance company equity portfolios are positively associated with the local preferences.

**Table 4:** Regression analysis of local equity preferences of insurance companies.

Variables	*Insurer Dummy*_*i*,*j*,*t*_		*Holding Ratio*_*i*,*j*,*t*_	
	(1)	(2)	(3)	(4)
*Local*_*i*,*j*,*t*_	0.5059***	0.3141***	0.4716***	0.3214***
	(7.16)	(4.25)	(6.28)	(4.05)
*SOE*_*i*,*j*,*t*_		0.2346***		0.2366***
		(4.56)		(4.43)
*Size*_*i*,*j*,*t*_		0.3820***		0.3560***
		(17.48)		(15.54)
*Leverage*_*i*,*j*,*t*_		-0.4493***		-0.3617**
		(-3.26)		(-2.57)
*Top1*_*i*,*j*,*t*_		0.3387**		0.0653
		(2.24)		(0.42)
*ROA*_*i*,*j*,*t*_		4.3941***		4.8820***
		(8.72)		(9.58)
*Growth*_*i*,*j*,*t*_		-0.0865***		-0.0782***
		(-2.92)		(-2.60)
*Dividend ratio*_*i*,*j*,*t*_		0.1225**		0.1073**
		(2.39)		(2.02)
*Free float*_*i*,*j*,*t*_		0.5124***		0.5112***
		(5.79)		(5.53)
*Risk taking*_*i*,*j*,*t*_		-4.5695***		-5.2155***
		(-6.54)		(-7.14)
*EM*_*i*,*j*,*t*_		-0.8627***		-0.8484***
		(-3.78)		(-3.61)
*Market index*_*i*,*j*,*t*_		-0.3554*		-0.3828**
		(-1.94)		(-1.98)
*GDP*_*i*,*j*,*t*_		0.1151**		0.1230**
		(2.40)		(2.45)
*Per capita GDP*_*i*,*j*,*t*_		0.0050		0.0058
		(0.05)		(0.06)
Constant	-4.2687***	-15.0010***	-5.0774***	-15.3819***
	(-17.58)	(-10.14)	(-21.17)	(-9.91)
region- (city level), industry-, and year- FE	√	√	√	√
observations	336,825	336,825	336,825	336,825

Columns 1 and 2 (Columns 3 and 4) of Table 4 present the results of regressions for Eq (1) by using *Insurer dummy* (*Holding Ratio*), respectively, as two forms of proxies for *Insurer*, which is described and defined in Section 3.2. Columns 1 and 3 are results of not including control variables, the regression coefficients of *Local* are 0.5059 (t = 7.16) and 0.4716 (t = 6.028), respectively, which are statistically significant and positive at the level of 1%. After adding control variables, as displayed in columns 2 and 4, the regression coefficients of *Local* are of 0.3134 (t = 4.25) and 0.3214 (t = 4.05), respectively, which are also statistically significant and positive at the level of 1%. The numbers in parentheses are t -statistics and standard errors clustered at the firm and year level. * p<0.1; ** p<0.05; *** p<0.01.

Besides, the coefficients for the control variables, i.e., *Size*, *ROA*, *Dividend ratio*, and *Free float*, are all significantly positive, as shown in Table 4, indicating that firms with larger scale, better operating performance, a higher proportion of dividend payout and tradable shares are more welcomed by insurance institutions to invest, and the shareholding ratio of insurance funds being their large shareholders is also higher. The coefficients for the other control variables, i.e., *leverage*, *Risk-taking*, *and EM*, are all significantly negative, indicating that firms with higher leverage levels, poorer risk-taking capability, and the lower degree to information transparency are not favored by insurance companies to invest, and the shareholding ratio is also low.

### 4.3. Insurance companies’ local equity preferences and performances

Table 5 demonstrates the regression results for hypothesis 2. Columns 1 and 2 are the regression results when *ROA1* and *ROA3* proxy the operating performance. Columns 3 and 4 are the regression results when *BHR1* and *BHR3* are used to proxy the performances of stock return. Most of the variables related to the current performance (*ROA*) will inevitably influence future performance (*ROA1* and *ROA3*). Therefore, to avoid the endogeneity problem, we delete the current *ROA* in the regression model of the future performance of the enterprise in Eq (2). From the OLS regression for Eq (2) of various forms of measures to proxy *perf* in Eq (2), we display that the coefficient estimates of *Local* are not statistically significant. Following the rationale for Pool et al. (2012) [10], who demonstrate that if investors overinvest stocks located nearby because of the information superiority they have to access more information, the local investing should deliver superior performances, while familiarity will not affect performance. Our empirical results present that the local stocks do not significantly outperform nonlocal stocks, even though equity managers indeed invest significantly more in stocks that are geographically close to them than do those faraway. Thus, our results verify hypothesis H2b while rejecting H2a, confirming that insurance companies don’t have real information about firms nearby. Equity managers decide portfolios based on familiarity, which is consistent with Ackert et al. (2005) [21] and Seasholes and Zhu (2010) [9], who demonstrate that local investing is not truly informed but suffers from home bias.

**Table 5:** Regression analysis of insurance companies’ local equity preferences and performances.

Variables	*ROA1*_*j*,*t*_	*ROA3*_*j*,*t*_	*BHR1*_*j*,*t*_	*BHR3*_*j*,*t*_
	(1)	(2)	(3)	(4)
*Local Shareholder*_*j*,*t*_	-0.0007	0.0010	-0.0037	-0.0077
	(-0.25)	(0.32)	(-0.19)	(-0.44)
*SOE*_*j*,*t*_	-0.0077***	-0.0091***	-0.0608***	-0.0468***
	(-3.04)	(-3.10)	(-4.25)	(-2.84)
*Size*_*j*,*t*_	0.0062***	0.0050***	-0.0192***	-0.0372***
	(4.69)	(3.34)	(-2.91)	(-4.68)
*Leverage*_*j*,*t*_	-0.1149***	-0.1165***	0.1869***	-0.0049
	(-12.35)	(-10.91)	(3.85)	(-0.09)
*Top1*_*j*,*t*_	0.0286***	0.0224**	0.0588	0.0038
	(3.33)	(2.37)	(1.41)	(0.08)
*ROA*_*j*,*t*_	-	-	0.7202***	0.1547
	-	-	(3.75)	(0.74)
*Growth*_*j*,*t*_	0.0080***	0.0068***	0.0005	-0.0037
	(4.52)	(3.34)	(0.03)	(-0.19)
*Dividend ratio*_*j*,*t*_	-0.0042	-0.0053	-0.0128	0.0185
	(-1.20)	(-1.30)	(-0.55)	(0.77)
*Free float*_*j*,*t*_	0.0128***	0.0196***	-0.0718**	-0.0207
	(2.61)	(3.85)	(-1.98)	(-0.65)
*Risk taking*_*j*,*t*_	-0.1189**	-0.0723	-0.7483**	-0.1148
	(-2.37)	(-1.55)	(-2.54)	(-0.47)
*EM*_*j*,*t*_	0.0210*	0.0170	0.0572	0.0933
	(1.77)	(1.39)	(0.61)	(1.25)
*Market index*_*j*,*t*_	0.0110	0.0096	0.0963*	0.1165**
	(1.15)	(0.83)	(1.85)	(2.30)
*GDP*_*j*,*t*_	0.0001	0.0019	-0.0004	0.0066
	(0.04)	(0.60)	(-0.03)	(0.44)
*Per capita GDP*_*j*,*t*_	-0.0089*	-0.0077	-0.0742***	-0.0648***
	(-1.89)	(-1.43)	(-3.01)	(-2.84)
constant	-0.0045	-0.0047	2.5381***	1.4991***
	(-0.11)	(-0.10)	(9.78)	(6.20)
region- (city level), industry-, and year- FE	√	√	√	√
observations	5,404	3,749	5,390	3,733
Pseudo.R2/R2	0.2369	0.2664	0.5644	0.3436

Columns 1 and 2 are the regression results for Eq (2) when *ROA1* and *ROA3* proxy the operating performance, respectively; Columns 3 and 4 are the regression results for Eq (2) when *BHR1* and *BHR3* are used to measure the performances of stock return, respectively. From various forms of measures to proxy *perf*, which is described and defined in Section 3.2, in Eq (2), we display that the coefficient estimates of *Local* are not statistically significant. The numbers in parentheses are t -statistics and standard errors clustered at the firm and year level. * p<0.1; ** p<0.05; *** p<0.01.

### 4.4. Additional analyses

Our results so far have suggested strong shreds of evidences against the information advantage presumption. In this section, we now test in what circumstance insurance companies are more prone to exhibit local equity preferences.

#### 4.4.1. Regional cultural differences

To obtain more insight into the effect of home bias for insurance companies on local investing, we next focus on whether differences in culture matter the degree to which insurance companies overweigh local portfolios. We test the “culture” characteristics mainly based on previous studies suggesting that culture values affect multiple aspects of fundamental economic decision-making [38, 39, 43–45]. Culture proximity exerts a profound influence on firms’ choice of overseas listing, which would make diversification gains exhausted [46]. Cultural proximity between debtors and creditors can mitigate information friction by reducing transaction costs, thus increase loan scale and improve loan quality [47]. This strand of literature sheds light on that national or regional culture differences contribute to forming unique social connections, social networks, and a sense of identification, which play a prominent role in firms’ financial decisions and probably provide new evidence for justifying why stay-at-home investments make sense.

Currently, China’s provincial administrative division segments the distribution of regional culture, giving rise to areas belonging to the same regional culture are divided into other provinces. For instance, the dialects spoken in Suzhou, Wuxi, and Changzhou in the south of Jiangsu Province are Wu dialect, making their cultural customs and codes of conduct closer to Zhejiang Province, which speaks Wu dialect, but the three cities are administratively classified into the Jiangsu Province. It is, thus, conceivable that these three dialect-segmented areas encounter cultural contradictions with the mainstream culture of Jiangsu province they administratively belong to. Chinese scholars Gao and Long (2016) use dialects as the proxy to measure the cultural differences, and they find cultural segmentation caused by administrative division lowers regional economic growth in China [48]. Therefore, to test the cultural characteristic affecting the local preferences in insurance companies, we refer to Gao and Long’s (2016) [48] method by using dialect to proxy culture differences and divide our research sample into two sub-sample groups. If the dialect of a certain city is the same as that of its provincial capital city, it means that the city’s culture is identical to its provincial mainstream culture (marked as group dialect-same below). In contrast, if the dialect of a certain city is different from that of its provincial capital city, it means that its culture is segmented from the mainstream (marked as group dialect-different below).

Column 1 and column 2 (Column 3 and column 4) in Table 6 presents the results of sub-sample regression for Eq (1) using *Insurer dummy* (*Holding Ratio*) as two forms of proxies for insurance companies’ equity investments, respectively. The coefficients of *Local* are both statistically significant and positive in dialect-different groups, as shown in columns 2 and 4, which suggests that the incentive driving the local preferences becomes stronger when the insurance companies come from culture-divided areas. Our result is consistent with the results report in Grinblatt and Keloharju (2001) [17], who show that firms that communicate with investors’ native tongue are more likely to be traded. We explain why familiarity in identity contributes to investor bias towards certain stocks as cultural differences give rise to mistrust and informal institutional conflicts, which increase transaction costs. When insurance companies from culturally divided areas invest elsewhere, investors become reluctant to hold stocks of firms with which they are not identical and familiar in principles of conduct, making home-based investments more to occur.

**Table 6:** Regional cultural differences and insurance companies’ local preferences.

Variables	*Insurer Dummy*_*i*,*j*,*t*_		*Holding Ratio*_*i*,*j*,*t*_	
	*dialect-same*	*dialect-different*	*dialect-same*	*dialect-different*
	(1)	(2)	(3)	(4)
*Local*_*i*,*j*,*t*_	0.1090	0.3504***	0.1061	0.3599***
	(0.56)	(4.15)	(0.53)	(3.95)
controls	√	√	√	√
region- (city level), industry-, and year- FE	√	√	√	√
observations	88,842	247,983	88,842	247,983

Columns 1 and 2 (Columns 3 and 4) of Table 6 present the results of sub-sample regressions for Eq (1) by using *Insurer dummy* (*Holding Ratio*), respectively, as two forms of proxies for *Insurer*, which is described and defined in Section 3.2. As displayed in columns 2 and 4, the regression coefficients of *Local* of 0.3504 (t = 4.15) and 0.3599 (t = 3.95), respectively, are both statistically significant and positive at the level of 1% in dialect-different groups. The numbers in parentheses are t -statistics and standard errors clustered at the firm and year level. * p<0.1; ** p<0.05; *** p<0.01.

#### 4.4.2. Heterogeneity of insurance companies

Prior studies have shown differences between property and life insurers in many aspects, e.g., product characteristics, capital sources, matching of asset-liability durations, *etc*. Property insurers are mainly committed to developing short-term insurance products, while life insurers are committed to long-term ones. In Table 7, we investigate whether differences in the business scope of insurers are associated with the degree to which insurance companies overweight local investing. We divide the full sample into two sub-samples based on insurers’ primary business scope: *life insurer* group and *non-life insurer* group.

**Table 7:** Differences in insurers’ business scope and insurance companies’ local preferences.

Variables	*Insurer Dummy*_*i*,*j*,*t*_		*Holding Ratio*_*i*,*j*,*t*_	
	*non-life insurer*	*Life insurer*	*non-life insurer*	*Life insurer*
	(1)	(2)	(3)	(4)
*Local*_*i*,*j*,*t*_	0.1815	0.3291***	0.1779	0.3385***
	(0.78)	(4.48)	(0.75)	(4.27)
Controls	√	√	√	√
region- (city level), industry-, and year- FE	√	√	√	√
observations	74,330	262,495	74,330	262,495

Columns 1 and 2/Columns 3 and 4 of Table 7 present the results of sub-sample regressions for Eq (1) by using *Insurer dummy* (*Holding Ratio*), respectively, as two forms of proxies for *insurer*, which is described and defined in Section 3.2. As displayed in columns 2 and 4, the regression coefficients of *Local* of 0.3291 (t = 4.48) and 0.3385 (t = 4.27), respectively, are both statistically significant and positive at the level of 1% in *life insurer* groups. The numbers in parentheses are t -statistics and standard errors clustered at the firm and year level. * p<0.1; ** p<0.05; *** p<0.01.

In the regression results for Eq (1) with *Insurer dummy* (*Holding Ratio*) as two forms of proxy for the explained variable, respectively, the estimates on *Local* are significant at 1% level and positive in both *life insurer* groups, as shown in column 2 and 4 of Table 7. This implies that the local equity preferences are stronger in life insurers’ equity portfolio decisions. Usually, life insurer capital has the characteristics of large amount, long investment cycle, and low-risk preference, making their corporate equity subject to high risk and volatility, while property insurance funds focus more on current income and stock liquidity. Even property insurers hold shares of firms as their major shareholders; this would be short-term. This is consistent with the literature that has documented that life insurers are subject to a more restrictive set of regulatory constraints than other segments of the insurance sector [49]. These restrictions limit the ability of insurance companies to invest large fractions of their portfolios in unfamiliar firms. Therefore, investing in more familiar firms is more conducive to life insurers controlling investment risks and stabilizing investment income.

### 4.5. Robustness tests

To ensure the reliability of the main conclusions, we mainly carry out the following robustness tests. We use two other alternative measures to replace *Local*: (i) *Local province*, which is a dummy variable equal to one if the insurance company and the stockholding firm are located in the same province, and zero if not; (ii) and *Ln (distance)* expressed as the natural logarithm of the linear distance between the insurance company and the latitude and longitude of its investee firm’s registration place. After including all controlled variables that are same as the Tables 4–7, Table 8 Panel A reports the results from estimating Eq (1), where columns 1 and 2 report results when *Insurer Dummy* proxies the insurance company equity holdings and columns 3 and 4 report results when this measure is proxied by *Holding Ratio*. The results of columns 1 and 3 presenting that the coefficient estimates of *Local province* are robust to measure geographical proximity at province-level, rather than at city level, implying the “local preference” for insurance companies in portfolios decision is stronger when companies and their holdings are located in the same province. The results of columns 2 and 4 show that the coefficients of *Ln (distance)* are both negative and statistically significant at the level of 1%, presenting that the incentive for insurance companies to invest in local firms becomes weaker when the distance between the companies and their holdings are farther. Moreover, we re-examine the results after excluding giant insurance companies in China, such as China Life, Ping An Insurance and China Pacific Insurance to confirm the robustness of the geographical effect, the results remain the same shown in Table 8 Panel B. The above pieces of evidence reinforce the validity of the main conclusions of our paper.

**Table 8:** Robustness tests.

Variables	Panel A				Panel B	
	*Insurer Dummy*_*i*,*j*,*t*_		*Holding Ratio*_*i*,*j*,*t*_		*Insurer Dummy*_*i*,*j*,*t*_	*Holding Ratio*_*i*,*j*,*t*_
	(1)	(2)	(3)	(4)	(5)	(6)
*Local province*_*i*,*j*,*t*_	0.2915***		0.2935***			
	(4.35)		(4.11)			
*Ln (distance)*_*i*,*j*,*t*_		-0.0599***		-0.0609***		
		(-4.27)		(-4.06)		
*Local*_*i*,*j*,*t*_					0.4756***	0.4928***
					(3.78)	(3.78)
Controls	√	√	√	√	√	√
region-, industry-, and year- FE	√	√	√	√	√	√
Observations	336,825	336,825	336,825	336,825	336,825	336,825

Column 1 and column 2 (Column 3 and column 4) of Table 8 present the results of regression for Eq (1) using *Insurer dummy* (*Holding Ratio*), respectively, as two forms of proxies for *insurer*, which is described and defined in Section 3.2. Columns 1 and 3 report regression results for Eq (1) in which the coefficients estimate of *Local province* were 0.2915 (t = 4.35) and 0.2935 (t = 4.11), respectively, both statistically significant and positive at the level of 1%. Columns 2 and 4 report regression results for Eq (1) in which the regression coefficients of *Ln (distance)* of -0.0599 (t = -4.27) and -0.0609 (t = -4.06), respectively, both statistically significant and negative at the level of 1%. Column 5 and column 6 of Table 8 present the results of regression for sub-sample that exclude giant insurance companies. The coefficients estimate of *Local* were 0.4746(t = 3.78) and 0.4928(t-3.78), respectively. The numbers in parentheses are t -statistics and standard errors clustered at the firm and year level. * p<0.1; ** p<0.05; *** p<0.01.

## 5. Conclusions and limitations

Using China’s data from 2006 to 2016, we identify that geographical proximity plays a prominent role in determining insurance companies’ equity portfolios choice. Although many scholars differ in explaining why investors prefer investing nearby, as we deliver in this paper, the geographical proximity does not produce real information to eliminating information asymmetry; however, home bias formed by familiarity affects insurers’ investment strategies. Gauged by examining the associations with whether the stocks of firms are headquartered the same with insurance companies and the equity holdings of insurance companies, we first find that insurance companies significantly tilt to invest in local firms geographically headquartered nearby; we then find that the local investments do not deliver superior performance than non-local investments. We additionally find that the local equity preference in insurance companies is stronger in areas with a high degree of dialect segmentation and life insurers. Such shreds of evidence collectively show that the preferences for investing nearby apply to equity portfolios of insurance companies and are more suitable to be explained by home bias, which may account for the low returns on equity investments of insurance funds.

Our paper suggests several promising directions for future research. Firstly, based on examining such highly regulated institutional investors as insurance companies, our research develops and puts forward empirical evidence that behavioral factors affect investor decision-making, which strengthens Huberman’s (2001) [16], Ackert et al.’s (2005) [21], and Pool et al.’s (2012) [10] evidence. Secondly, our results showing that local investments do not deliver excellent performance metrics fill the research blank for how professional managers generate investment strategies. To promote the sustainable and high-quality development of the non-bank financial institutions as the world’s second-largest institutional investors, insurers’ equity managers should devote themselves to optimize their equity investment allocations to improve in-vestment return, instead of produce less-sophisticated investment strategies due to hometown bias to impair investor’s benefits. Unfortunately, due to limited data availability, we are unable to obtain detailed information on insurance company fund investments, making it difficult to find direct evidence to indicate that insurance fund managers process financial information of local firms worse than non-local firms. Future research is suggested to focus on the details of insurance fund investment in listed companies. We also encourage further research to analyze whether consumer taste affects the investment decisions of insurance portfolio managers when data is available.
